# IL-6 Promotes Hepatocellular Carcinoma Invasion by Releasing Exosomal miR-133a-3p

**DOI:** 10.1155/2022/4589163

**Published:** 2022-04-06

**Authors:** Xudong Ren, Yu Zhou, Yunling Luo, Chaoqun Wang, Anna Pan, Yanqin Ju, Haoting Sun, Zhifei Lin, Beiyuan Hu, Guangzheng Sun, Wenwei Zhu, Liang Hong

**Affiliations:** ^1^Department of General Surgery, Huashan Hospital, Fudan University, Shanghai, China; ^2^Department of Infectious Diseases, The Third Affiliated Hospital of Wenzhou Medical University, Wenzhou, China; ^3^Department of Stomatology, Huashan Hospital, Fudan University, Shanghai, China; ^4^Department of General Surgery, The Third Affiliated Hospital of Wenzhou Medical University, Wenzhou, China

## Abstract

Interleukin-6 (IL-6), an important inflammatory cytokine, is a key factor regulating cancer metastasis. Cancer cells can modulate their tumorigenic abilities by sorting specific microRNAs (miRNAs) as exosomes into the tumor microenvironment. The relationship between IL-6 and exosomal miRNAs related to hepatocellular carcinoma (HCC) metastasis remains to be elucidated. We examined the metastatic ability of HCC cells after IL-6 treatment and found that miR-133a-3p was sorted into exosomes after IL-6 stimulation and was subsequently released into the tumor microenvironment. In vitro analysis confirmed that exosomal miR-133a-3p acted as a tumor suppressor in HCC. Bioinformatic analysis revealed several signaling pathways and hub genes (CREB1, VCP, CALM1, and YES1) regulated by miR-133a-3p. Survival curves further verified the important roles of hub genes in the prognosis of patients with HCC. It is envisaged that the IL-6/miR-133a-3p axis may be related to the activation of CREB1, VCP, CALM1, and YES1. Our findings provide new insights into the role of exosomal miRNA-mediated tumor progression under inflammatory conditions.

## 1. Introduction

Hepatocellular carcinoma (HCC) is one of the most prevalent tumors with high malignancy. The curative effect is still poor owing to the high metastasis and recurrence rates of HCC [1]. Therefore, finding an efficient way to control the metastasis is crucial, and understanding the mechanism of HCC is of vital importance.

Interleukin-6 (IL-6) is a critical mediator of HCC development [2] and is involved in the regulation of cancer stem cell proliferation, differentiation, invasion, metastasis, and angiogenesis through a number of signaling pathways, such as STAT3 signaling, which drives cancer progression and metastasis [3]. Increased serum levels of IL-6 are found in patients with viral and alcoholic hepatitis and liver cirrhosis, which are the risk factors for the development of HCC [4, 5]. Elevated IL-6 serum levels were correlated with an increased risk of developing HCC [3]. Our previous work has also demonstrated that IL-6 plays a pivotal role in HCC metastasis by upregulating osteopontin (OPN) [6].

Exosomes are nanovesicles (30–150 nm) released from various sources, such as cells and body fluids, into the microenvironment [7, 8] and transfer mRNAs, microRNAs (miRNAs), and proteins to recipient cells or tissues [9]. miRNAs are noncoding RNAs that regulate the target gene expression and further modulate the metastasis of malignant tumors, including HCC [10, 11]. Our group has previously demonstrated that miR-26a could suppress the tumor growth and metastasis of HCC through IL-6/STAT3 signaling [12]. However, IL-6 is also known to enhance the metastatic ability of HCC cells [6]; whether this process is related to exosomal miRNAs and the underlying mechanism remain unclear.

In the present study, we found that MHCC-97L cells with low metastatic potential exhibited enhanced invasive and metastatic abilities by releasing exosomes containing tumor suppressor miR-133a-3p upon IL-6 treatment. Through bioinformatic analysis, we identified the molecular regulatory network of miR-133a-3p, which is hypothesized to control tumor metastasis and recurrence.

## 2. Materials and Methods

### 2.1. Cell Culture

HCC cells with low metastatic (MHCC-97L, HepG2, and Hep3B) or high-metastatic (HCCLM3 and MHCC-97H) potential were cultured as previously described [6]. Briefly, cells were cultured in Dulbecco's modified Eagle's medium (DMEM) (Hyclone, Logan, UT, United States) supplemented with 10% fetal bovine serum (FBS) (Gibco, U.S.A.) and maintained in a humidified incubator with 5% CO_2_ at 37°C.

### 2.2. Vectors and Transfection

miR-133a-3p mimic and inhibitor oligos as well as the corresponding negative control were synthesized by RiboBio (Guangzhou, China). HCC cells were seeded in 6-well plates and cultured overnight for transfection. Transfection was performed according to the manufacturer's instructions.

### 2.3. Isolation and Identification of Exosomes

Cells were seeded in dishes to achieve confluency of 80%, and then, fresh culture medium was added. After culturing for another 48 h in the culture medium with exosome-depleted FBS (Gibco, U.S.A.), the medium was collected and used for exosome isolation. The supernatant was collected into a 50 ml centrifuge tube, centrifuged for 15 min at 4°C and 4000 × g to remove cell debris, and then filtered through a 0.22 *μ*m membrane filter to further remove other components. The supernatant was further filtered using a Millipore 100 kD column and centrifuged at 4°C and 4000 × g for 30 min. Phosphate-Buffered Saline (PBS) was then added to the column, and it was centrifuged at 4000 × g for 30 min to remove phenol red and other impurities. The previous step was repeated, and PBS was added again followed by a subsequent washing step. The concentrated supernatant was used for electron microscopy and subsequent experiments. The lower filtrate was collected and centrifuged using a Millipore 10 kD column at 4°C and 4000 × g for 30 min. A concentrated supernatant, which did not contain exosomes, was obtained and was used as a blank control. The exosomes were detected by the presence of CD9 and CD63 markers (Cell Signaling Technology) using western blotting, and the morphology of the exosomes was visualized by transmission electron microscopy (TEM). The size distribution of these exosomes was examined using NanoSight Tracking Analysis (Malvern NanoSight NS300, UK).

### 2.4. Wound Healing, Migration, and Invasion Assays

For the wound healing assay, cells were seeded into 6-well plates to achieve confluency of 80%. An artificial wound was then created in the confluent cell monolayer of the cells. Photographs were taken at 0, 24, and 48 h using an inverted microscope (Carl Zeiss, Inc., Thornwood, NY, U.S.A.). Migration and invasion assays were conducted in Transwell chambers (Costar, Corning Inc., NY, USA) coated with or without Matrigel (BD Biosciences) on the upper surface of the 8 *μ*m (pore size) membrane. Briefly, HCC cells treated with or without 50 ng/ml IL-6 for 48 h were harvested, suspended in serum-free medium, and plated into the upper chamber for the migration or invasion assays, respectively; media supplemented with 10% FBS were placed into the lower chamber. After 24 h of incubation, the cells that had migrated or invaded through the membrane to the lower surface were fixed, stained, and counted using a microscope.

### 2.5. Cell Proliferation Assay

Cells in the logarithmic growth phase were digested with trypsin, neutralized with 1 ml of new complete medium, resuspended, and counted, and the concentration of the cell suspension was adjusted to 10^4^ cells/ml. The cells were seeded in 6-well plates at a density of 3 × 10^4^ cells/well and cultured in a cell incubator at 37°C. DMSO or IL-6 were added to the plates. The cells were digested and counted every 24 h.

### 2.6. Real-Time PCR

Reverse transcription was carried out according to the instructions provided with the PrimeScript™ RT reagent Kit (Takara), and SYBR Premix Ex Taq II (Takara) was used for real-time PCR. Gene expression of miR-133-3p was determined with primers: forward 5′-TGGTCCCCTTCAACCAG-3′ and reverse 5′-GGTCCAGTTTTTTTTTTTTTTTCAG-3′.

### 2.7. Bioinformatic Analysis

RNA extraction from the cells and exosomes was performed as described previously [13, 14]. The miRNA microarray was performed by KangChen Bio-tech Inc. (Shanghai, China). GEO dataset GSE40367 was analyzed using GEO2R to identify differentially expressed genes (DEGs) between primary and metastatic HCC. The target genes of miR-133a-3p in HCC were identified using TargetScan (https://www.targehttp://tscan.org/vert_72/). Hub genes were identified using Venn overlap between DEGs and miR-133a-3p target genes (http://bioinformatics.psb.ugent.be/webtools/Venn/). A protein-protein interaction (PPI) network was established by integrating protein information from the Search Tool for the Retrieval of Interacting Genes (STRING) database (http://string-db.org/). The Database for Annotation, Visualization, and Integrated Discovery (DAVID) online platform (https://david.ncifcrf.gov/) was used to analyze the hub genes. The Gene Expression Profiling Interactive Analysis (GEPIA) database (http://gepia.cancer-pku.cn/index.html) was used to generate survival curves and determine overall survival (OS) rates and their correlation with the hub genes in HCC [15].

### 2.8. Statistical Analysis

Data were analyzed using SPSS 22.0 software (IBM). The measurement data are expressed as *X* ± *S*. One-way ANOVA and LSD method were used for comparisons among the groups. The *t*-test was used for pairwise comparisons. The test level was set at *α* = 0.05.

## 3. Results

### 3.1. IL-6 Can Promote the Invasive Ability of HCC Cells

**Figure 1:**
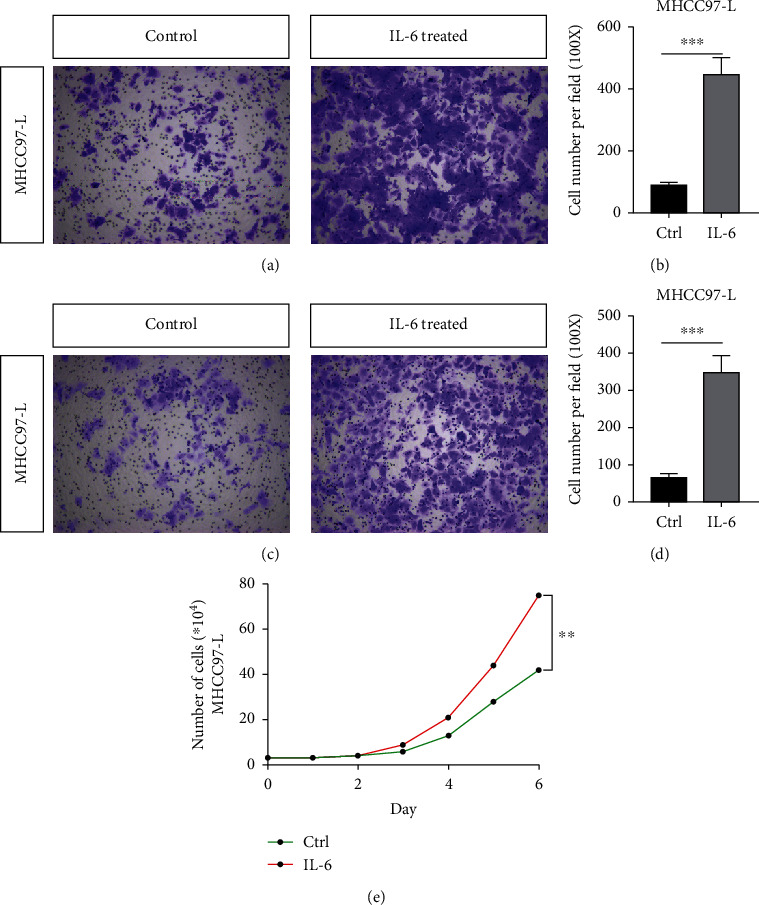
IL-6 can promote the invasion and metastasis of HCC. (a) The migration ability of MHCC-97L cells with/without IL-6 treatment. (b) Statistical analysis explaining the images presented in (a). (c, d) The invasion ability of MHCC-97L cells. (e) The growth curve showing improved proliferation of MHCC-97L cells after IL-6 treatment. ^∗∗∗^*p* < 0.01, *n* = 5. Ctrl: control group.

The migration ability of MHCC-97L cells was significantly improved after IL-6 treatment (Figures 1(a) and 1(b)). IL-6 treatment also enhanced the invasion ability of MHCC-97L cells (Figures 1(c) and 1(d)). The growth curve showed that the proliferation of MHCC-97L cells was significantly improved after IL-6 treatment. The results showed that IL-6 could significantly enhance the invasion, metastasis, and proliferation of MHCC-97L cells with low metastatic potential.

### 3.2. MHCC-97L Cells Treated with IL-6 Gain the Ability of Invasion by Releasing Exosomes

**Figure 2:**
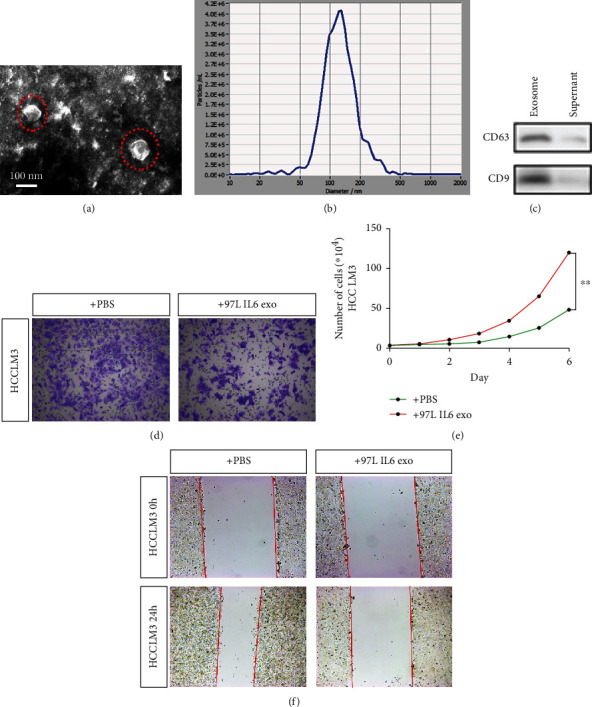
IL-6-treated MHCC-97L cells obtain the ability of invasion and metastasis by releasing exosomes. (a) Transmission electron micrograph of exosomes, scale bar 100 nm. (b) The size of exosomes detected by NTA is consistent with the previously reported size of 30-150 nm. (c) Detection of CD63 and CD9 in exosomes. (d) Transwell invasion assay showed the invasion ability of HCCLM3 cells. (e) Graph representing the number of cells passed through in the invasion assay. (f) The callus ability of HCCLM3 cells as detected by the scratch test. ^∗^*p* < 0.05, *n* = 3.

To investigate the effect of IL-6 on exosomes secreted by MHCC-97L cells, exosomes were harvested from the supernatants of IL-6 pretreated MHCC-97L cells via a gradient centrifugation method, as described previously [16]. The cup-shaped structure of exosomes was observed by transmission electron microscopy (TEM) (Figure 2(a)). The size of the extracted exosomes detected by NTA was consistent with their reported size of 30–150 nm (Figure 2(b)) The specific expression of CD63 and CD9 in the supernatant also confirmed that the extracted components were indeed the exosomes derived from MHCC-97L cells (Figure 2(c)). To further study whether the exosomes released by IL-6 pretreated MHCC-97L cells (97L-IL6-exo) contain certain molecules that can inhibit the invasion and metastasis of tumor cells, we added the extracted exosomes into the HCC cell line (HCCLM3) with high-metastatic potential. The Transwell invasion assay showed that HCCLM3 cells with 97L-IL6-exo had weaker invasion ability (Figure 2(d)), and the number of cells traversed was significantly reduced (Figure 2(e)). In the wound healing assay, the callus ability of HCCLM3 cells with 97L-IL6-exo also decreased significantly (Figure 2(f)). These results illustrated that the exosomes released by MHCC-97L cells after IL-6 treatment could significantly inhibit the invasion, metastasis, and proliferation of tumor cells with high-metastatic potential.

### 3.3. miR-133a-3p in Exosomes Can Inhibit the Invasion and Metastasis of Tumor Cells

**Figure 3:**
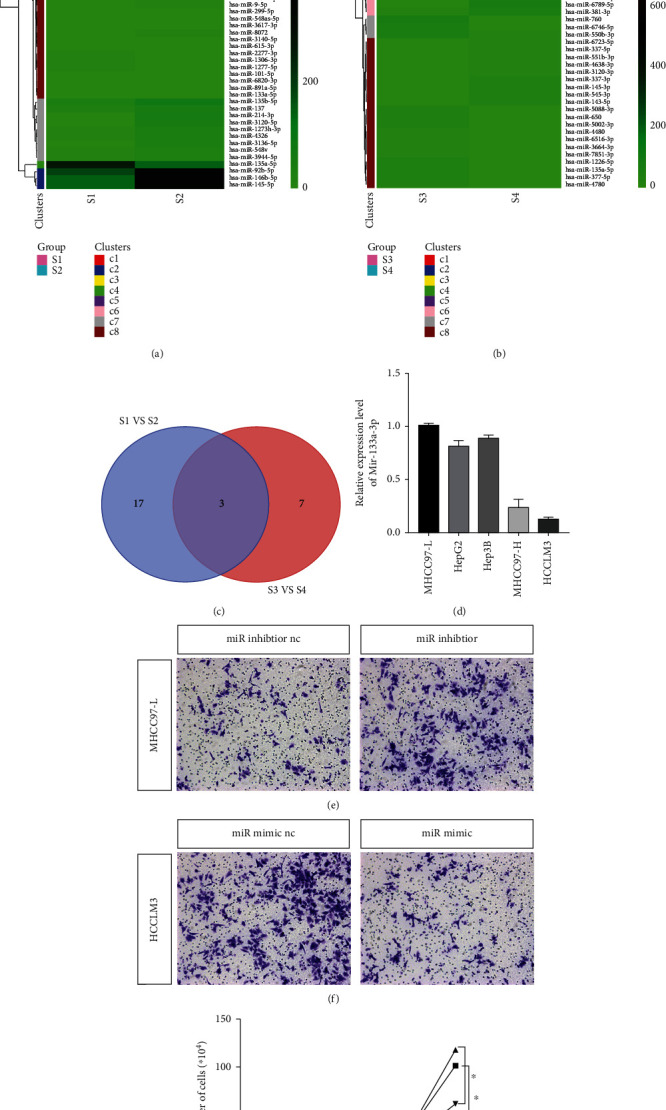
miR-133a-3p in exosomes can inhibit the invasion and metastasis of tumor cells. (a, b) The cluster heat map demonstrates the differentially expressed miRNAs. Rows represent miRNAs while columns represent tissues. S1 and S2 represent the miRNAs of MHCC-97L cells with/without IL-6 treatment, while S3 and S4 represent the exosomal miRNAs of MHCC-97L cells with/without IL-6 treatment, respectively. (c) Overlapping expression miRNAs in different groups is illustrated using a Venn diagram. (d) Real-time PCR showing the relative expression of miR-133a-3p in different HCC cell lines. (e, f) Transwell assay showing the invasive ability of MHCC-97L and HCCLM3 cells upon treatment with miR-133a-3p inhibitor and mimic, respectively. (g) The effect of miR-133a-3p inhibitor and mimic on the proliferation of HCC cells. ^∗^*p* < 0.05, *n* = 3. Nc: negative control.

To identify the factors that can inhibit tumor invasion and metastasis of cells treated with 97L-IL6-exo, exosomal miRNAs and cellular miRNAs were detected using miRNA microarray analysis. Twenty differentially expressed miRNAs that displayed fold changes > 2 (*p* < 0.05) compared to the levels observed in the MHCC-97L cells group were identified in the IL-6 pretreated MHCC-97L cells (Figure 3(a), Table 1). Ten differentially expressed miRNAs were identified in the exosomes released from IL-6 pretreated MHCC-97L cells as compared with those identified in the exosomes released from untreated MHCC-97L cells (Figure 3(b), Table 2). The distributions of the differentially expressed miRNAs and their overlapping expression in these different groups are illustrated using a Venn diagram in Figure 3(c), which shows that the levels of 3 miRNAs (miR-133a-3p, miR-199a-5p, and miR-143-3p) were significantly changed in both cells and exosomes after IL-6 treatment. The expression level of miR-133a-3p decreased in MHCC-97L cells but increased in the exosomes after IL-6 treatment, indicating that MHCC-97L cells may have released the miR-133a-3p into the cytoplasm through exosomes after IL-6 treatment.

Subsequently, the expression levels of miR-133a-3p in different HCC cell lines were detected using qRT-PCR. The results showed that the expression level of miR-133a-3p was negatively correlated with the metastatic potential of the cells (Figure 3(d)). Further, the miR-133a-3p inhibitor was added to MHCC-97L cells, which increased their invasive ability (Figure 3(e)). When miR-133a-3p mimics were added to HCCLM3 cells, the invasive ability of these cells was found to have been reduced (Figure 3(f)). Similar results were obtained in the cell proliferation experiments (Figure 3(g)). These results showed that HCC cells with low metastatic potential could exhibit invasion and metastasis potential by releasing exosomes containing miR-133a-3p after IL-6 treatment. miR-133a-3p is known to be an important tumor suppressor [17, 18].

### 3.4. Bioinformatic Analysis Revealed the Hub Genes of IL-6/miR-133a-3p Axis

**Figure 4:**
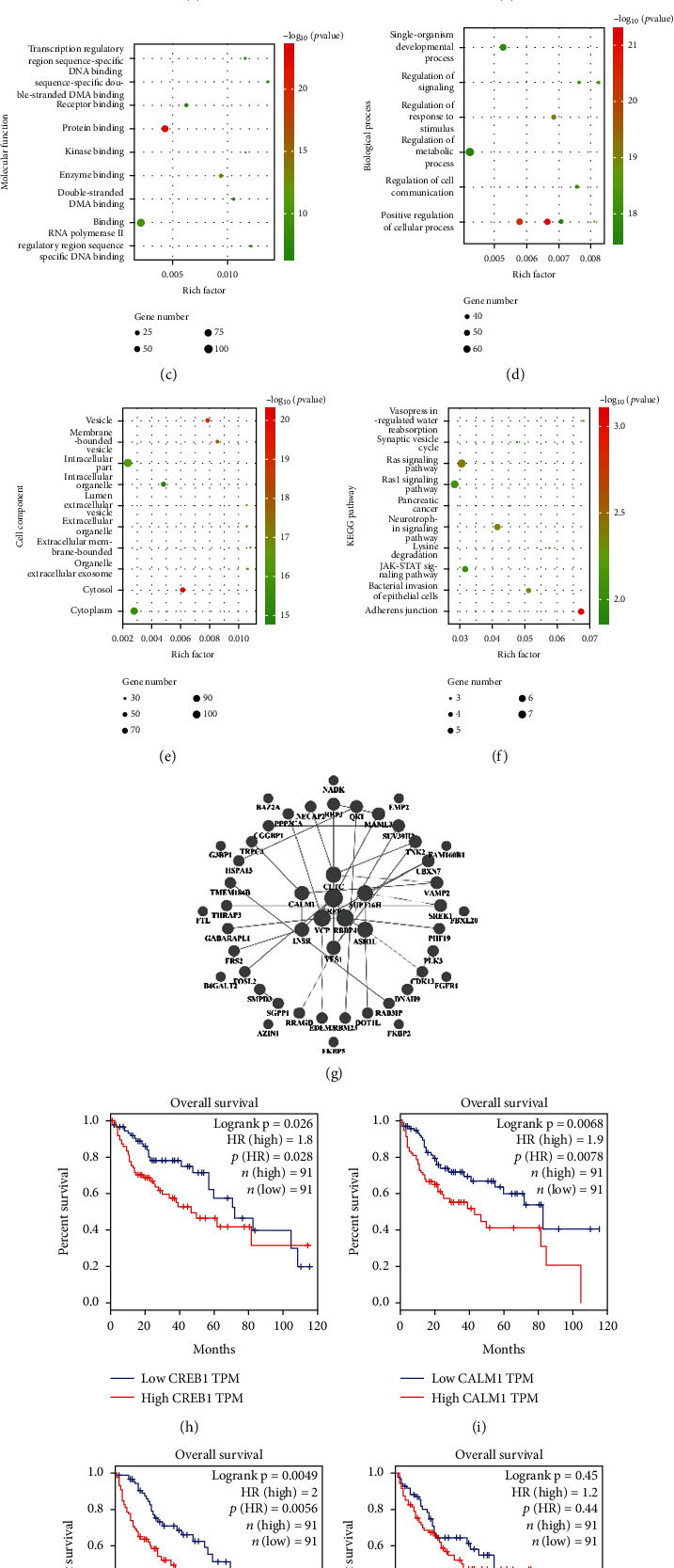
Bioinformatic analysis reveals the hub genes of IL-6/miR-133a-3p axis. (a) Identification of 3973 differentially expressed genes (DEGs) by comparing the metastatic HCC with nonmetastatic HCC based on the GSE40367 database. The volcano plot demonstrates the fold changes and statistical significance. (b) Venn diagram illustrating the overlapping expression genes between 3973 DEGs and 571 target genes. GO analysis of the 140 overlapping expression genes indicating (c) molecular functions (MF), (d) biological processes (BP), and (e) cellular components (CC). (f) KEGG pathway analysis. (g) PPI network analysis reveals the hub genes. (h–k) Overall survival analysis of the hub genes by Kaplan-Meier plots. The *p* values were calculated using log-rank statistics.

Next, we explored the molecular regulatory network of miR-133a-3p as well as its target genes that control tumor metastasis and recurrence. We found 3973 differentially expressed genes (DEGs) by comparing the metastatic HCC with primary HCC based on the GSE40367 database and used a volcano plot to identify fold changes and statistical significance (Figure 4(a)). A total of 571 downstream target genes of miR-133a-3p were identified using the TargetScan web server. We then analyzed 3973 DEGs and 571 target genes and identified 140 overlapping expression genes, which are illustrated using a Venn diagram in Figure 4(b). Gene Ontology (GO) analysis revealed that these 140 genes were mainly related to molecular binding in various cellular functions (Figure 4(c)) and to the regulation of metabolism under physiological function (Figure 4(d)). The cell composition analysis showed that the target gene functions were mainly distributed in the intracellular locations and cytoplasm (Figure 4(e)). Kyoto Encyclopedia of Genes and Genomes (KEGG) pathway enrichment analysis indicated that these genes may be involved in the RAS, Rap1, and JAK-STAT pathways (Figure 4(f)). Based on PPI network analysis, we identified nine hub genes, among which CREB1, VCP, CALM1, and YES1 were highly expressed in the metastatic group (Figure 4(g)). Therefore, it can be concluded that HCC cells with low metastatic potential acquire invasive and metastatic potential by releasing exosomes containing miR-133a-3p after IL-6 stimulation, which may be related to the activation of CREB1, VCP, CALM1, and YES1. Moreover, we investigated the prognosis of CREB1, CALM1, VCP, and YES1 activation using GEPIA databases [15] in HCC. Increased expression levels of CREB1, CALM1, VCP, and YES1 were associated with poor overall survival (OS) in HCC, which further verified that these genes can mediate HCC metastasis (Figures 4(h)–4(k)).

## 4. Discussion

Metastasis has now been recognized as an important process in cancer progression [19]. IL-6 plays an important role in the recurrence and metastasis of HCC. Several studies have reported that HCC patients with significantly high plasma IL-6 levels are at a high risk of postoperative tumor recurrence [20]. Our previous work also showed that plasma IL-6 levels were positively correlated with postoperative tumor recurrence and tumor thrombus in microvessels [6, 12]. IL-6 can enhance stemness and epithelial-mesenchymal transition (EMT) in HCC cells [6]. Consistent with the previous studies, our results also confirmed that IL-6 can promote the invasion and metastasis of HCC cells. In this study, we evaluated the underlying mechanism of IL-6-mediated metastasis of HCC.

Exosomes, small extracellular vesicles released from the cells and body fluids, carry miRNAs that have various biological functions. miRNAs, as noncoding RNAs, participate in many biological processes by regulating their target gene expression [21, 22]. Several researchers have found that miRNAs as tumor suppressors can influence carcinogenesis, including that of HCC [23, 24]. Growing evidence has shown that miR-133a is downregulated and plays tumor suppressor roles in cancer and that it can inhibit proliferation, migration, and invasion of HCC cells by targeting several genes such as IGF-1R, FSCN1, and FOSL2 through the TGF-*β*/Smad3 signaling pathway [17, 18]. Consistent with these previous studies, our results showed that the expression level of miR-133a-3p was decreased in IL-6 preconditioned MHCC-97L cells and increased in the exosomes released from these cells, which indicated that MHCC-97L cells released miR-133a-3p into the cytoplasm through exosomes after IL-6 stimulation. In addition, our results also verified that the expression level of miR-133a-3p was negatively correlated with the ability of cell metastasis and invasion and that miR-133a-3p is an important tumor suppressor in HCC.

Interestingly, the other two microRNAs (miR-199a-5p and miR-143-3p) were also significantly changed in both cells and exosomes after IL-6 treatment. Exosomal miR-199a-5p was found to play an important role in hepatic lipid accumulation [25]. Our previous work has also found that exosomal miR-143-3p might upregulate MARCKS in tumor-associated macrophages and was associated with immune infiltration [26]. Wozniak et al. demonstrate that inflammasome-mediated RILP cleavage influences sequence-specific miRNA loading into exosomes via interactions with unique cargo vesicles and association with RNA binding proteins [27]. As an inflammation-related tumor, the occurrence of liver cancer is accompanied by a large number of inflammatory components, including IL-6. Therefore, IL-6 might regulate ESCRT (endosomal sorting complex required for transport) in HCC cells and load specific microRNAs (miR-133a-3p/miR-199a-5p/miR-143-3p) into exosomes. The microRNAs released from HCC cells could also be taken in by other cells in the tumor microenvironment like macrophages, endothelial cells, and tumor-associated fibroblasts for more functions such as immune escape [28] and angiogenesis [29].

Furthermore, based on the PPI network analysis, four hub genes (CREB1, VCP, CALM1, and YES1) that may be related to the IL-6/miR-133a-3p axis were identified. An investigation regarding the association between the gene expression levels and survival rate further verified that these genes may mediate HCC metastasis. CREB1 (cAMP response element-binding protein 1) belongs to the basic leucine zipper domain class of proteins. It binds to the cAMP response element of its target genes to regulate gene transcription [30]. CREB1 has been shown to enhance HCC progression by supporting angiogenesis and rendering HCC cells resistant to apoptosis [31]. A previous study has shown that CREB1 was directly inhibited by miRNAs in HCC, and knockdown of CREB1 by siRNAs impeded the migration and invasion of MHCC-97H cells [32]. VCP (valosin-containing protein), also known as p97, belongs to the AAA family (ATPase with multiple cellular activities). It has been reported that the level of VCP is associated with the prognosis of many types of carcinomas [33]. Studies have illustrated that the expression of VCP is directly regulated by miRNAs (such as miR-129-5p) [34] and is correlated with an increased incidence of HCC recurrence [35]. CALM1 (Calmodulin 1) is a ubiquitous calcium ion (Ca^2+^) receptor protein, which is composed of Ca^2+^-binding EF-hands, and participates in signaling pathways that regulate proliferation, motility, and differentiation [36]. Several studies have found that the expression level of CALM1 is markedly associated with many types of cancer [37]. YES1 tyrosine kinase has been identified as the cellular homologue of the oncogenic viral yes gene product [38] and has been implicated in a variety of signaling pathways, including growth factor responses, alterations in the cytoskeleton, cell cycle progression, apoptosis, and differentiation [39]. Recent studies have shown that upregulation of miR-210 could inhibit the proliferation of HCC cells by targeting YES1, and miR-133a-3p could bind the 3′UTR of YES1 and downregulate its expression [40, 41]. Survival analysis indicated that HCC patients with high expression levels of the hub genes suffered from poor prognosis. Still, the specific regulation mechanisms for these hub genes in HCC need further study.

In conclusion, we found that IL-6 can promote the invasion and metastasis of HCC cells by releasing exosomes containing miR-133a-3p. Bioinformatic analysis showed that this process may be related to the activation of CREB1, VCP, CALM1, and YES1. Targeting the IL-6/miR-133a-3p axis may play an important role in controlling the HCC metastasis.

## Figures and Tables

**Table 1:** Significant differential expression of miRNAs in MHCC-97L cells with/without IL-6 treatment.

MicroRNA	S1 normalized	S2 normalized	Log2FC	*p* value	Up-/downregulated
hsa-miR-214-3p	1.70	62.55	5.20	<0.001	Up
hsa-miR-9-5p	0.00	10.42	20.00	0.001	Up
hsa-miR-299-5p	0.00	7.36	20.00	0.018	Up
hsa-miR-3120-5p	1.13	53.35	5.56	<0.001	Up
hsa-miR-3591-3p	0.57	18.40	5.02	<0.001	Up
hsa-miR-199a-5p	0.57	11.05	4.29	0.008	Up
hsa-miR-122-5p	1.70	18.40	3.44	0.001	Up
hsa-miR-3940-3p	2.26	14.72	2.70	0.039	Up
hsa-miR-143-3p	37.35	219.53	2.56	<0.001	Up
hsa-miR-1255a	3.96	20.85	2.40	0.014	Up
hsa-miR-3615	4.53	22.08	2.29	0.016	Up
hsa-miR-3136-5p	7.92	33.11	2.06	0.004	Up
hsa-miR-3617-5p	44.14	128.16	1.54	<0.001	Up
hsa-miR-146b-5p	152.78	382.64	1.32	0.001	Up
hsa-miR-145-5p	152.78	366.08	1.26	0.003	Up
hsa-miR-451a	66.77	24.53	-1.44	0.022	Down
hsa-miR-133a-3p	94.49	32.50	-1.54	0.002	Down
hsa-miR-206	42.44	11.65	-1.86	0.005	Down
hsa-miR-1-3p	806.32	195.00	-2.05	<0.001	Down
hsa-miR-2277-3p	11.88	0.61	-4.28	0.006	Down

**Table 2:** Significant differential expression of exosomal miRNAs in MHCC-97L cells with/without IL-6 treatment.

MicroRNA	S3 normalized	S4 normalized	Log2FC	*p* value	Up-/downregulated
hsa-miR-133a-3p	192.50	545.17	1.50	0.040	Up
hsa-miR-376-3p	45.29	324.25	2.84	0.019	Up
hsa-miR-4634	56.62	488.16	3.11	0.004	Up
hsa-miR-376b-3p	4.85	62.36	3.68	0.025	Up
hsa-miR-219a-1-3p	4.85	65.92	3.76	0.017	Up
hsa-miR-199a-5p	27.50	427.59	3.96	<0.001	Up
hsa-miR-143-3p	50.15	1177.65	4.55	<0.001	Up
Has-miR-1207-5p	9.71	269.02	4.79	<0.001	Up
hsa-miR-6790-3p	0.00	51.67	20.00	0.001	Up
hsa-miR-337-3p	0.00	26.72	20.00	0.043	Up

## Data Availability

GEO dataset GSE40367 was analyzed using GEO2R to identify differentially expressed genes (DEGs) between primary and metastatic HCC. The target genes of miR-133a-3p in HCC were identified using TargetScan (https://www.targehttp://tscan.org/vert_72/). Hub genes were identified using Venn overlap between DEGs and miR-133a-3p target genes (http://bioinformatics.psb.ugent.be/webtools/Venn/). A protein-protein interaction (PPI) network was established by integrating protein information from the Search Tool for the Retrieval of Interacting Genes (STRING) database (http://string-db.org/). The Database for Annotation, Visualization, and Integrated Discovery (DAVID) online platform (https://david.ncifcrf.gov/) was used to analyze the hub genes. The Gene Expression Profiling Interactive Analysis (GEPIA) database (http://gepia.cancer-pku.cn/index.html) was used to generate survival curves and determine overall survival (OS) rates and their correlation with the hub genes in HCC.
